# Sedentariness and Back Health in Western Cape Primary School Students: Protocol for a Pragmatic Stepped-Wedge Feasibility Randomized Controlled Trial

**DOI:** 10.2196/18522

**Published:** 2020-11-30

**Authors:** Dominic Fisher, Quinette Louw, Lehana Thabane

**Affiliations:** 1 Division of Physiotherapy, Department of Health and Rehabilitation Sciences Faculty of Medicine and Health Sciences Stellenbosch University Cape Town South Africa; 2 Department of Health and Rehabilitation Sciences Stellenbosch University Cape Town South Africa; 3 Department of Health Research Methods, Evidence, and Impact (formerly the Department of Clinical Epidemiology and Biostatistics) McMaster University Hamilton, ON Canada; 4 Departments of Anesthesia and Pediatrics McMaster University Hamilton, ON Canada; 5 Biostatistics Unit St Joseph's Healthcare Hamilton, ON Canada; 6 Population Health Research Institute Hamilton Health Sciences Hamilton, ON Canada

**Keywords:** sitting, standing, postural dynamism, sit-stand desks, classroom, school, sedentary, children

## Abstract

**Background:**

Despite growing evidence of deleterious health outcomes associated with sedentary behavior, prolonged static sitting in classrooms remains ubiquitous in primary schools. Sedentary behavior is associated with the development of cardio-metabolic conditions and poor back health. Preventative strategies to reduce sedentary behavior and its negative health effects may be required in a resource-constrained environment such as South Africa.

**Objective:**

The primary objective of this study is to assess the feasibility of conducting a full trial to evaluate the effects of a multifaceted intervention comprising novel multifunctional classroom furniture and a video-based curriculum versus usual care on sedentary behavior among students aged 10-11 years in primary schools. The secondary objective is to assess the preliminary effects of the intervention on sedentary behavior and postural dynamism.

**Methods:**

Eighty grade 5 or 6 students, aged 10 and 11 years, in mixed-gender schools within the Western Cape metropolitan urban area in Cape Town, South Africa are eligible to participate in this pilot cluster stepped-wedge trial design with classroom as the unit of randomization. Data will be collected at the schools. The intervention will comprise multifunctional classroom furniture that allows for sitting and standing as well as a video-based curriculum on sedentary behavior. Usual practice is the absence of the intervention. The primary outcomes assessed will be (1) adherence to the intervention and (2) project pragmatics. The secondary outcomes will be (1) sedentariness measured using activPAL3 microsensors and (2) postural dynamism measured using Noraxon Myomotion inertial measurement units. We randomized the school to the first or second start of the intervention. This is an open-label trial and therefore blinding will not be possible for any group. Descriptive analysis of the feasibility and physiological outcomes will be presented. We will report the preliminary estimates of the effects of the intervention on sedentariness and postural dynamism using the mean difference and 95% CI.

**Results:**

At the time of submission, two classrooms have been recruited into the study. Baseline physical activity and postural dynamism data have been collected from 10 participants from each class.

**Conclusions:**

The results of this feasibility stepped-wedge cluster randomized controlled trial will be useful in informing the design of the main trial to assess whether this multifaceted intervention of multifunctional classroom furniture that allows for sitting and standing as well as a video-based curriculum versus usual care has any effect on sedentary behavior in low-resource-setting primary schools.

**Trial Registration:**

Pan African Trials Registry PACTR201811799476016; https://tinyurl.com/y4upoys8

**International Registered Report Identifier (IRRID):**

RR1-10.2196/18522

## Introduction

### Background

A lifestyle of sedentary behavior may result in the development of noncommunicable conditions such as metabolic syndrome [1] and poor back health [2]. Metabolic syndrome represents a range of interrelated disorders comprising abdominal obesity, raised blood pressure, dyslipidemia, and hyperglycemia [1]. Even when the guidelines for physical activity are met, sedentariness remains an independent risk factor for the development of noncommunicable diseases (NCDs) [3].

The school environment typically requires students to remain seated for prolonged periods of time, thus encouraging sedentary behavior [4]. Prolonged periods of sitting with accentuated thoracic kyphosis reduces and even reverses the natural, protective spinal curvature [5]. Although the etiology of back pain is multifactorial, poor postural alignment and reduced postural dynamism (number and extent of body movements while sitting) are common risk factors of back pain [6-9]. Static sitting may place an excessive physiological load on the spinal structures, leading to accumulative microdamage and consequent pain [10]. Dynamic posture reduces the accumulated damage from prolonged periods of sitting by breaking up the sitting periods into smaller epochs, which may help to reduce back pain [11] owing to a reduction in accumulated intervertebral disc compression [12]. Therefore, modifying the design of school furniture to optimize postural alignment and dynamism during sitting and increasing sit-stand transitions may address the potential health risks caused by prolonged sitting in the school environment. However, most of the research aimed at testing the efficacy of dynamic classroom furniture to increase classroom physical activity has been conducted in high-income settings [11,13-24]. Moreover, there is a paucity of studies regarding the effectiveness of classroom furniture in reducing classroom sedentariness and promoting back health, especially for low-to-middle income countries (LMICs). Currently, no back health intervention studies have examined an association with sedentary behavior, despite its recognition as an important risk factor. Furthermore, the majority of studies have implemented unimodal interventions to address either back pain or NCDs separately. Designing health programs that address more than one health problem is preferable in LMICs where health budgets are constrained. To our knowledge, no studies have addressed sedentariness by assessing the effect of a multimodal approach aimed at reducing sedentariness and promoting back health.

Prior to embarking on a large, definitive trial to assess a multimodal intervention aimed at reducing classroom sedentariness, an imperative first step is conducting a feasibility study [25]. Feasibility studies provide information on whether an intervention is contextually viable [26]. Therefore, the primary aim of this project is to assess the feasibility of conducting a multimodal, contextualized classroom-based intervention aimed at reducing sedentariness in primary school students. The secondary project aim is to yield preliminary data on the effect of the intervention on physiological outcomes of interest, namely physical activity and postural dynamism.

### Study Aims and Objectives

#### Primary Objectives

Adherence and project pragmatics are the two primary feasibility outcomes of interest in this study. Adherence to the intervention refers to the extent to which students and teachers comply with the novel furniture and utilize the health education video curriculum. The pragmatics refer to effectively capturing physical activity and postural dynamism data with activPAL microsensors (PAL Technologies, Glasgow, UK) and Noraxon Myomotion (Noraxon, Scottsdale, USA) inertial measurement units (IMUs), respectively. Several pragmatic factors have been identified that could threaten data loss, such as electromagnetic interference from classroom furniture and building infrastructure, Wi-Fi frequency traffic from wireless-enabled devices in close proximity, and IMU sensor battery performance during multiple days of capturing prolonged trials in excess of 1 hour. To our knowledge, activPAL sensors have not previously been used to measure physical activity in this population in South Africa. This study therefore provides the first opportunity to appraise acceptability of using this technology.

#### Secondary Objectives

The secondary study objectives are (1) to determine the preliminary effects of the intervention on sedentariness, and (2) to develop a novel method of analysis of postural data collected using IMUs in a real-life context.

## Methods

### Study Design

This study protocol, designed according to the CONSORT (Consolidated Standards of Reporting Trials) statement for feasibility trials [25], has a stratified, closed cohort, two-cluster stepped-wedge design with a pragmatic approach. The stepped-wedge design offers an ideal structure that allows for a robust evaluation of an intervention within the bounds of logistical constraints [27]. In addition, this study design allows both clusters to be exposed to the intervention with consideration of the unique context of each cluster.

### Participants

Two primary schools were recruited for participation in the study. Participant classrooms (children aged 10-11 years) will be the unit of randomization and both classrooms (clusters) will be exposed to the intervention but will serve as their own control. All participants in each cluster will be exposed to the intervention from which a sample will be selected to provide baseline and follow-up measurements, and qualitative interviewing. Measurements for each selected participant will be taken at baseline, prior to the onset of intervention implementation, and followed up at the end of the trial period. Baseline measurements and implementation of the intervention in classroom 2 will follow those of classroom 1. This study was granted ethical approval by Stellenbosch University Health Research Ethics Committee (reference number: S17/08/130).

### Study Intervention

The proposed intervention has two components: (1) novel height-adjustable sit-stand desks (KUZE desks; patent reference: A2017/01821), and (2) a series of health educational videos that encourage reduced classroom sedentariness and promote classroom physical activity. Usual classroom furniture will be replaced with KUZE desks after baseline measurements are performed. Prior to the implementation of the intervention, the researcher will train the students and teachers on how to safely and effectively use the KUZE desks. The training will include how to select and adjust the seat and desk height for sitting and standing. When used as a standing desk, the KUZE desk is placed on the existing desk and adjusted to the appropriate height.

During a teacher consultation prior to the onset of the project, the teacher will be shown the playlist of short health education videos provided on a mobile external hard drive. The videos are voiceover animations about the importance of reducing classroom sedentariness, awareness of good sitting posture, and increasing physical activity beyond the classroom. All videos contain an interactive segment of physical activity linked with simple mathematics tasks that the students are encouraged to engage with. The frequency at which the videos should be played will not be prescribed to the teacher. Strategies based on findings from a prior qualitative process that explored the potential barriers and facilitators to implementing a classroom-based intervention in primary school classes will be discussed.

### Setting

This study will be conducted in two primary school classrooms in the Western Cape region of Cape Town, South Africa. The Western Cape is characterized by broad socioeconomic, language, and cultural diversity, which will allow for the feasibility of the intervention to be tested in a resource-constrained environment as well as in an adequately resourced school environment. If the study is deemed to be feasible in a challenging resourced-strained environment, we consider that it will also be feasible in a range of less challenging scenarios.

### Trial Design

**Figure 1:**
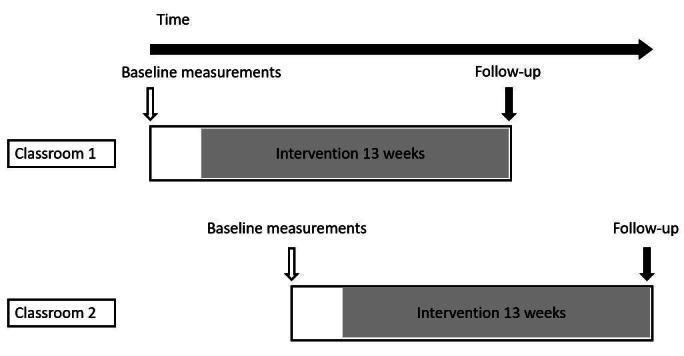
Study design.

This is a school-based, pilot cluster stepped-wedge feasibility trial. Individual students in grade 5 or 6 (10-11 years old) will be the unit of analysis with the classroom as the unit of randomization. Quintiles are socioeconomic categorizations of schools in South Africa that determine the state funding of the school, which is based on income levels, dependency ratios, and literacy rates in the area [28]. Although there will be no need to stratify the randomization of the clusters in this trial as there are only two participant classrooms, it is proposed that stratification by school quintile should be carried out during a future full trial. Baseline physiological data will be measured prior to the start of the intervention. Interviews with teachers and focus group discussions will be conducted after the intervention to assess the feasibility of the study. Figure 1 shows the proposed study design, and Multimedia Appendix 1 provides a SPIRIT diagram [29] for the design and timescales of the feasibility study.

### Feasibility Outcomes

Two broad feasibility criteria categories are to be considered: adherence and project pragmatics. The detailed success criteria and indicators are outlined in Table 1.

**Table 1:** Feasibility criteria.

Feasibility criteria	Success indicator	Measurement method
Delivery of the health education videos	The teacher develops a suitable routine of playing the videos and adheres to it	Qualitative feedback at exit interview (at 13weeks)
Acceptance of KUZE as a sit-stand desk	Students and teachers accepted the KUZE as the classroom furniture for the entire study period	Qualitative feedback at exit interview (at 13 weeks)
Compliance with wearing activPAL and IMU^a^ sensors	100% of the subsample selected to be monitored agreed and complied to wearing the sensors during the data collection sessions	Researcher monitoring dropout rate
Usage of the KUZE desk	15% reduction in sitting time, reduction in episodes of prolonged sitting, increase in standing time, increase in sit-to-stand transitions	activPAL
Classroom retention to the study	All classrooms initially recruited remain in the study	Researcher monitoring
Integrity of physical activity and postural dynamism data	The integrity (degree of corrupt data due to technical or human factors) of 80% of all data captured with objective tools	Researcher monitoring

^a^IMU: inertial measurement unit.

### Interpretation of Feasibility Success Criteria

Following the conclusion of the project, the success criteria will be interpreted as follows: (i) continue with large pilot/feasibility trial if all 6 success criteria are met, (ii) make minor modifications to the protocol if 3 or more criteria are met before continuing with a pilot/trial, and (iii) make significant protocol modifications if only 1 criterion is met [25].

### Physiological Measures

Classroom sitting will be measured with activPAL3 microsensors attached to the anterior right thigh as prescribed by the user manual. The outcomes of interest will include total classroom sitting time, standing time, and sit-to-stand transitions. The activPAL3 microsensor will be placed on the participants prior to the start of the school day and removed at the end of the school day before they are dismissed home. The data captured during recess times will be excluded from the data analysis. The activPAL3 microsensor has been increasingly used to objectively measure physical activity in recent years owing to its user-friendliness, practicality, and validity [30].

Postural dynamism will be measured using Noraxon Myomotion IMUs, which combine triaxial gyroscopes, accelerometers, and magnetometers. IMUs placed on the head, neck, thorax, and sacrum will be able to produce 3D kinematic and temporal outcomes, which can then be processed to produce a postural dynamism outcome.

### Sample Size

A sample size calculation was not performed as per the CONSORT extension statement for feasibility studies [25]. A classroom from a fee-paying and a nonfee-paying school will be recruited. The diversity within the sample is expected to provide a sufficiently broad spectrum of considerations to assess the feasibility of the intervention and the pragmatics of data collection. To limit the impact on the classroom environment, no more than 10 morning data collection sessions per cluster are planned. The availability of only 2 Noraxon systems and 10 activPAL sensors presents a logistical constraint that will only allow for the collection of postural dynamism data and classroom physical activity data from 20 students from each cluster respectively. The sample selected for measuring physiological outcomes will be randomly chosen from the class list. An equal number of males and females of 10 to 11-year-old students will be sampled. The participants in the qualitative study will include students who took part in physiological data collection and those who were only exposed to the intervention but did not provide physiological data.

### School Recruitment and Inclusion Criteria

Publicly funded schools will be stratified by socioeconomic categorization and then randomly contacted from a list of schools provided by the Western Cape Education Department. Only schools that have the requisite infrastructure to conduct the intervention will be considered for inclusion. This infrastructure includes technology for broadcasting the videos during normal teaching time and noninclined classroom desks. Schools that are conducting programs aimed at reducing classroom sedentariness or promoting spinal health will be excluded. At least one school from a predominantly black community will be recruited. A single class from the participant school that has students aged 10 to 11 years will be recruited into the study (Multimedia Appendix 1). Informed, written child assent and parent consent will be sought from the class teacher.

### Participant Recruitment and Inclusion Criteria

All students in the class (regardless of age) will be provided with a project information document, along with assent and parent/guardian consent forms, which will explain the methods and intervention as well as the researchers’ details. All students who return the completed assent and consent forms will be exposed to the intervention, but measurements will only be obtained from a sample of students.

### Process Evaluation

The process will be evaluated through deductive content analysis of transcribed interview data collected during qualitative in-depth interviews with the two school principals and two class teachers as well as focus group discussions with the students from each participant class. Topics related to the process of negotiating access to the classroom, impact of the experimental setup for the collection of data, acceptability of and fidelity to the intervention, impact of the intervention in the classroom, and monitoring of the implementation of the intervention will be discussed.

### Physiological Outcomes

Measurements will be taken from the study sample from each classroom at baseline and after implementation of the intervention to assess its effect. Classroom sitting will be measured for 10 consecutive school days using the activPAL3 microsensor. The sensor will be wrapped in a waterproof nitrile sleeve and attached to the proximal anterior right thigh of the participant with Hydrofilm. Participants will be required to wear the sensor from the beginning of the school day and it will be removed and collected by the researcher by the end of the school day.

### School and Participation Appreciation

As an expression of appreciation to participating schools, they will be offered up to five KUZE desks as well as the video series for ongoing use. The staff, students, and their parent/guardians will also be invited to a presentation of the relevant findings of the study.

### Data Analysis

#### Statistical Analysis

As per the CONSORT statement [25], feasibility outcomes will be described descriptively and narratively. Descriptive statistics will be used to report the clinical endpoints related to the feasibility outcomes. The number of participant classrooms and students, the number of children that remain in the study, and how many children use the school furniture throughout the study will be summarized. Owing to the small sample size, inferential statistics will most likely not be applicable. Study acceptability data will be presented per cluster as well as for the whole sample.

#### Qualitative Analysis

Thematic content analysis will be performed using Atlas.ti computer software by identifying and coding main themes and subthemes from the transcripts. A coding template will be compiled from the emerging themes and topics of interest relevant to the study objectives. An analytical diary will be kept alongside this coding template for justification of emerging themes. The coding template will be refined as more transcripts are coded. Encoded themes will be placed in context for analysis to recognize the significance and to understand the meaning thereof. Data will be analyzed per target group and by themes in cases of overlapping data between groups.

## Results

At the time of submission, two classrooms have been recruited into the study. Baseline physical activity and postural dynamism data have been collected from 10 participants from each class.

## Discussion

### Projected Outcomes

Sedentary behavior, typified by prolonged periods of sitting, is not only increasingly associated with the development of cardio-metabolic disorders [31,32] but is also associated with adverse spinal changes [5]. Considering that the lifetime prevalence of back pain among adolescents in Africa is 36% [33] and that back pain experienced during adolescence often progresses to chronic pain during adulthood [34], there is significant potential to improve the health of many South Africans and reduce the health burden by effective health promotion. In a country with a significant health burden as a result of infectious diseases such as HIV/AIDS and tuberculosis, health promotion should be prioritized to avoid preventable health costs.

Preventative programs aimed at reducing adverse health outcomes are well-aligned with the South African Department of Health’s agenda [35]. Its current policy to reintegrate and strengthen health care in schools attempts to improve access to early interventions and screening to a large proportion of the population with poor access to health care. It goes without saying that the efficacy of health programs must be rigorously tested through well-designed and efficiently conducted trials. A near essential preliminary step before conducting a full-scale trial is ensuring its feasibility [26].

Sit-stand classroom furniture has been shown to be effective in reducing classroom sedentary behavior [36] in several studies in well-resourced settings. However, no studies to test the effects of sit-stand classroom furniture on health outcomes have been previously conducted in South Africa. The resource-constrained South African context necessitates conducting a feasibility study ahead of a large, expensive trial. This feasibility trial will provide invaluable design and roll-out information for a future full-scale efficacy study.

### Conclusion

This study protocol will provide methodological detail and contextual insights of the proposed feasibility trial findings. Researchers planning on conducting a stepped-wedge trial of the effectiveness of novel multifunctional classroom furniture and video sedentariness curriculum on student sedentary behavior in South African primary schools will find helpful guidance from this protocol.

## Appendix

Multimedia Appendix 1 SPIRIT checklist.

## Abbreviations

CONSORT: Consolidated Standards of Reporting Trials
IMU: inertial measurement unit
LMIC: low-to-middle income country
NCD: noncommunicable disease
