# Supplementary figures and images for: Sedentariness and Back Health in Western Cape Primary School Students: Protocol for a Pragmatic Stepped-Wedge Feasibility Randomized Controlled Trial
Source: JMIR Res Protoc. 2020 Nov 30;9(11):e18522. doi: 10.2196/18522 (PMC7735899; doi:10.2196/18522)

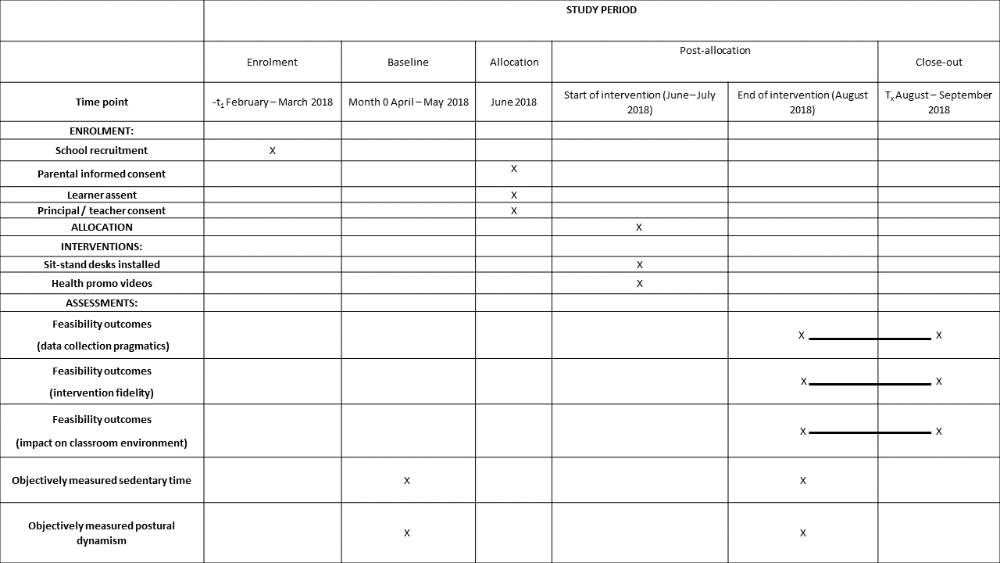

Supplement: Multimedia Appendix 1 [file resprot_v9i11e18522_app1.png]
